# Regenerative abilities of mesenchymal stem cells through mitochondrial transfer

**DOI:** 10.1186/s12929-018-0429-1

**Published:** 2018-03-30

**Authors:** Swati Paliwal, Rituparna Chaudhuri, Anurag Agrawal, Sujata Mohanty

**Affiliations:** 1 Stem Cell Facility, DBT Centre of Excellence for Stem Cell Research, All India Institute of Medical Sciences, New Delhi, 110029 India; 2 Molecular Immunogenetics Laboratory and Centre of Excellence for Translational Research in Asthma & Lung disease, CSIR-Institute of Genomics and Integrative Biology, Delhi, 110007 India

**Keywords:** Mesenchymal stem cells, Mitochondrial transfer mechanism, Regenerative medicine

## Abstract

The past decade has witnessed an upsurge in studies demonstrating mitochondrial transfer as one of the emerging mechanisms through which mesenchymal stem cells (MSCs) can regenerate and repair damaged cells or tissues. It has been found to play a critical role in healing several diseases related to brain injury, cardiac myopathies, muscle sepsis, lung disorders and acute respiratory disorders. Several studies have shown that various mechanisms are involved in mitochondrial transfer that includes tunnel tube formation, micro vesicle formation, gap junctions, cell fusion and others modes of transfer. Few studies have investigated the mechanisms that contribute to mitochondrial transfer, primarily comprising of signaling pathways involved in tunnel tube formation that facilitates tunnel tube formation for movement of mitochondria from one cell to another. Various stress signals such as release of damaged mitochondria, mtDNA and mitochondrial products along with elevated reactive oxygen species levels trigger the transfer of mitochondria from MSCs to recipient cells. However, extensive cell signaling pathways that lead to mitochondrial transfer from healthy cells are still under investigation and the changes that contribute to restoration of mitochondrial bioenergetics in recipient cells remain largely elusive. In this review, we have discussed the phenomenon of mitochondrial transfer from MSCs to neighboring stressed cells, and how this aids in cellular repair and regeneration of different organs such as lung, heart, eye, brain and kidney. The potential scope of mitochondrial transfer in providing novel therapeutic strategies for treatment of various pathophysiological conditions has also been discussed.

## Background

Recent advancements in regenerative medicine have capitalized on using stem cells for cellular repair and regeneration. Mesenchymal stem cells (MSCs) have emerged as promising candidates to treat various diseases owing to their unique characteristic properties such as self-renewal, immuno-suppressive potential and ability to trans-differentiate. They are easily available from various tissue sources such as bone marrow, adipose tissue, dental, umbilical cord etc. with low risk of rejection and several clinical trials have provided evidences of their beneficial roles. Interestingly, apart from through engraftment, MSCs are also being shown to exert their actions through paracrine mechanisms, thus repairing cellular damage [1–3]. These paracrine effects through which MSCs mediate repair can be through release of several immuno-modulatory factors, microvesicles, microRNAs, exosomes and mitochondrial transfer [4]. Mitochondrial dysfunction is associated with a majority of degenerative diseases like ischemic heart diseases, lung disorders, stroke, brain injury and several degenerative diseases including cardiomyopathy, Parkinson’s and Alzheimer’s [5–7]. Thus, mitochondrial transfer alone has appeared as a great therapeutic strategy as it can restore the bioenergetic needs of damaged cells. The transfer of healthy mitochondria appears to be an effective reparative strategy to rejuvenate several kinds of damaged cells such as epithelial cells, endothelial cells, cardiomyocytes and cells of other origins as well. In this review article, we will be discussing the role of mitochondrial transfer through MSCs and how it aids in repair and regeneration of injured and damaged cells under stress.

### Discovery of mitochondrial transfer and its impact on regenerative medicine

The transfer of mitochondria, which is a DNA containing organelle, is quite an intriguing phenomenon. Initial discovery of this process was observed in a co-culture system when mitochondrial DNA from donor human MSCs was found in recipient cells devoid of mitochondria [8]. Human epithelial cells when treated with ethidium bromide for long duration lost their mitochondrial activity. These human epithelial cells devoid of mitochondria (A549 rho cells) were co-cultured with healthy MSCs. It was observed that mitochondria respiration of epithelial cells could be restored when co-cultured with MSCs through mitochondrial transfer. This was demonstrated by presence of DsRed2 tagged mitochondria in MSCs and observed by time-lapse photo microscopy. It was also confirmed that uptake of mitochondria was facilitated by active and not passive transfer [8]. Dysfunctional mitochondria can lead to cellular damage and apoptosis, thus a rescue mechanism wherein healthy mitochondria can be transferred has shown tremendous potential. Other cells such as fibroblast and somatic cells have also shown the ability to transfer mitochondria. Mitochondrial donation by MSCs is a faster and more economical physiological process in the cell to replace dysfunctional mitochondria in diseased cells/tissue, as compared to the mitochondrial biogenesis and thus, appears as an efficient means to attenuate a disease conditions. During the time that Spees et al. published their work, many research groups had simultaneously acknowledged and identified the formation of tunnel tube formation for cell-cell communications [9–12]. Rustom et al.*,* had demonstrated de novo formation of multiple tunnel tubes leading to complex networks between cells and facilitating transfer of membrane vesicles and organelles between cells [9]. These tunnel tubes comprise of F-actin based connections between distant cells and exist in diverse morphologies carrying multiple cargos and signals between cells [13]. These studies were followed by a plethora of studies that provided evidence for mitochondrial transfer between MSCs and damaged cells of varied origins [14–18]. The discovery of remarkable mitochondrial transfer ability of MSCs to cells with dysfunctional mitochondria paved way for numerous studies [14]. MSCs from different tissue sources like bone marrow, adipose, and Wharton’s jelly have now been shown to transfer mitochondria to various damaged cells, like osteosarcoma cells, that aid in the restoration of their respiratory activities [19]. Table 1 provides a list of studies performed using different sources of stem cells in different conditions, described in detail in later sections of the review.

**Table 1:** Mitochondrial transfer from Different Tissue Specific MSCs to Recipient Cells of Different Origins

S. No	MSCs Type	Recipient Cell	Mode of Transfer	Action	References
1	BM-MSCs	A549 Cell Line	Cellular Contact and Cytoplasmic Projections	Restored Aerobic Respiration	[8]
2	BM-MSCs	Pulmonary Alveoli in mouse lungs	Connexin43 alveolar attachment and microvesicles	Protection against acute lung injury	[15]
3	BM-MSCs and iPSCs	Airway epithelial cells	Tunnel Tubes	Rescue cigarettesmoke induced damage	[20]
4	BM-MSCs	Bronchial Epithelial Cells	Tunnel Tubes and Miro1	Rescue Bronchial Epithelial Injury	[21]
5	BM-MSCs	H9c2 cardiomyocyte cell line	Tunnel Tubes	Rescue cardiomyocytes in ischemia model	[22]
6	BM-MSCs	Cardiomyocytes	Tunnel Tubes	Repair damaged cardiomyocytes in failed myocardium	[23]
7	BM-MSCs	Cardiomyoblasts	Cell-Cell Connection and Tubular Connections	Rescue Cells from Ischemic Damage	[24]
8	BM-MSCs	Kidney Tubular Epithelial cells	Microvesicles	Recovery from acute kidney injury	[25]
9	BM-MSCs	Rat Cortical Neuronal Cells	Tunnel Tubes and Miro1	Neuroprotective	[26]
10	BM-MSCs	Macrophages	Tunnel Tubes	Anti-Bacterial Activity	[27]
11	BM-MSCs	Macrophages	Extracellular Vesicles	Regulation of mitochondrial dynamics and Mitophagy	[28]
12	BM-MSCs	Acute myeloid leukemic cells	Cell-Cell Contact	Survival of Myeloid Cells	[18]
13	BM-MSCs	MDA-MB231 breast carcinoma cell line	Isolated mitochondria	Enhanced OXPHOS activity	[29]
14	iPSCs-MSCs	Corneal Epithelial Cells	Tunnel Tubes	Protection of corneal epithelial cells against alkali burn	[16]
15	AD- MSCs	Cardiomyocytes	Cell Fusion	Reprogram adult cardiac cells towards progenitor like state	[30]
16	WJ-MSCs	Cells devoid of mitochondria Osteosarcoma cells	Tunnel Tubes	Mitochondria function was rescued	[19]

### Mitochondrial bioenergetics status in MSCs

**Fig. 1:**
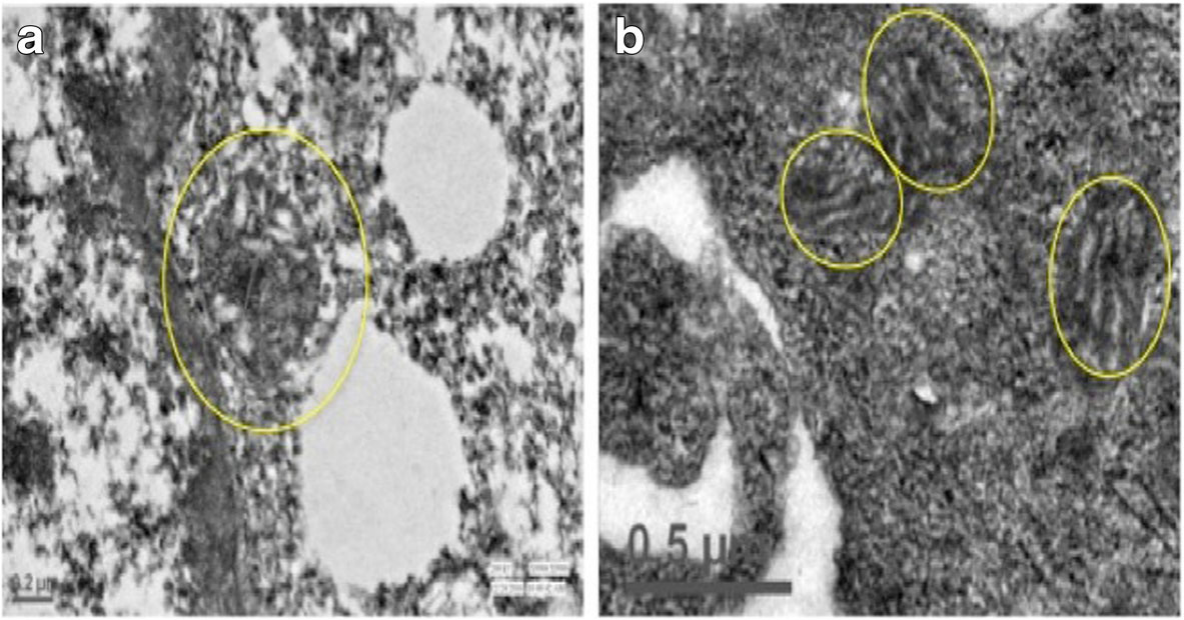
Striking difference between mitochondria of Human MSCs and JEG-3 Cell Line as observed under Transmission Electron Microscope (TECNAI 200 Kv, Fei, Electron Optics). **a** Human BM-MSCs cells: Mitochondria are fewer in number, spherical, condensed with underdeveloped cristae and display cytoplasmic localization. Scale Bar: 0.2 μm (**b**) JEG-3 Cell Line, mitochondria are branched, tubular, elongated and well-organized cristae, more in number and peri-nuclear in localization. Scale Bar: 0.5 μm

Mitochondria play important roles in oxidative phosphorylation, ATP generation, and cellular apoptosis. At the mitochondrial inner membrane, electron transport chain is involved in the generation of energy, where oxygen acts as the electron acceptor. During oxidative phosphorylation, electron leak from mitochondrial complexes in electron transport chain results in partial reduction of oxygen and generation of reactive oxygen species (ROS). Antioxidant enzymes present in the cells metabolize toxic intermediates to maintain cellular homeostasis. However, these control mechanisms are not effective in dysfunctional mitochondria and accumulation of ROS results in oxidative damage in cell. In a bio-energetically active cells, increased ROS production triggers dynamic changes in mitochondria and mitochondrial fragmentation occurs through fission and autophagy. Under stress, these cellular mechanisms are unable to maintain mitochondrial homeostasis resulting in dysfunctional mitochondria. In contrast, MSCs maintain themselves in the glycolytic state, and switch from glycolysis to oxidative phosphorylation upon differentiation to meet energy needs of the cells. Mitochondria in MSC exist in dormant state and display low activity status due to lesser energy demands. However upon differentiation, an increase in mtDNA copy number, high levels of respiratory enzymes and mRNA levels, enhanced oxygen consumption rate and increased levels of intracellular ATP are observed [20, 21]. Extensive restructuring and remodeling of the mitochondria occurs during differentiation or transfer to cells with higher bioenergetics demands. It is well established that mitochondrial biogenesis and aerobic respiration gets unregulated in MSCs during differentiation and oxidative stress. Mitochondrial biogenesis is controlled by proliferator activated receptor gamma coactivator-1 α (PGC-1 α). This further activates nuclear respiratory factors (NRF 1 and NRF 2) and mitochondrial transcription factors (mTFA) which coordinates DNA polymerase γ and facilitates replication of mtDNA [22]. This is followed by concomitant increase in intracellular ATP levels and up regulation of oxidative metabolism in MSCs. A complex regulatory network of proteins maintains cellular homeostasis between mitochondrial biogenesis and cellular metabolism of MSCs. This process is further tightly regulated by fission, fusion and mitophagy that impact its shape, size and location in the cell [23–25]. Studies provide evidence that mitochondrial metabolism, cell differentiation, stemness of stem cells and cellular apoptosis are interdependent cellular processes [24, 26–29]. On the basis of energy needs ranging from low to high, mitochondrial shape varies from small, fragmented, circular, undeveloped cristae to larger networks of elongated mitochondria with well-developed cristae, respectively. This transformation eventually impacts mitochondrial number, morphology, cellular metabolism and mitochondrial activity [6, 20, 30, 31]. Stem cells maintain low levels of cellular ATP, low levels of oxidative damage levels and tightly regulated redox balance as opposed to differentiated cells [20, 26, 32]. In Fig. 1, we have shown mitochondrial status in transmission electron images of human BM-MSCs and human choriocarcinoma cell line JEG-3, to demonstrate a striking difference in the mitochondria of two cells with low and high-energy demands respectively (unpublished data). The mitochondria in MSCs are spherical in shape with underdeveloped cristae, cytoplasmic localization and less in number, owing to lower energy demand (Fig. 1a). In contrast, cell lines, here JEG-3, have higher energy and thus, branched, elongated mitochondria with well-organized and localized in peri-nuclear region (Fig. 1b). These naive mitochondria are transferred from MSCs to cells with dysfunctional mitochondria to with high oxidative energy demands thereby modulating their cellular metabolism. Mitochondria transfer appears to be a lucrative strategy for regenerative processes, as mitochondrial biosynthesis usually take longer than can be afforded by damaged cells in crisis. Thus, mitochondrial transfer is an efficient method for regeneration of injured cells. Although complete mechanisms of mitochondria transfer have not been elucidated yet, but certain studies suggests that mitochondria transfer from MSCs modulate cellular metabolism of recipient cells. Up regulation of mitochondrial respiration and ATP levels along with reduction in oxidative damage has been observed in several studies wherein MSCs have been co-cultured with injured cells or tissues [16, 33–35]. It is plausible that mitochondria transferred from MSCs modulate cellular bioenergetics by regulating mtDNA replication, maintaining its copy number and regulating other mitochondrial dynamics and cellular processing required for maintenance of mitochondrial homeostasis in cell.

### Signals that trigger mitochondrial transfer from mesenchymal stem cells

The signals that trigger transfer of mitochondria from MSCs have intrigued scientists for a long time. Researchers have postulated theories suggesting that oxidative stress of dysfunctional mitochondria of recipient cell sends environment cues to the MSCs [15, 34]. The local microenvironment of an injured cell has been shown to release physiological cues that trigger transfer of mitochondria. It is compelling to note that bidirectional mitochondrial transfer occurs between MSCs and their microenvironment. Few studies have illustrated that stress signals released by recipient cells include damaged mitochondria, released mtDNA and mitochondrial products that are characterized as damage associated molecular patterns (DAMPS) [36–39]. Interestingly, mtDNA released by injured cells are engulfed by MSCs that subsequently triggers the cytoprotective function of MSCs and enhanced mitochondrial biogenesis through retrograde signaling, thereby preparing MSCs for mitochondrial donation [40]. In addition, reactive oxygen species released by cells under oxidative stress and inflammation status have also been postulated to trigger donation of mitochondria [15, 34, 41, 42]. Elevated levels of reactive oxygen species have been found in cells with high energy demands such as in cancer cells, and these usually have been seen to receive mitochondria from surrounding cells to meet its energy demands [43, 44]. In acute myeloid leukemic cells, NADPH oxidase-2 derived superoxide has been reported to stimulate mitochondrial transfer from BM-MSCs through tunnel tube formation [45]. As discussed previously, that co-culturing MSCs with cells under oxidative stress reversed the oxidative stress state by decreasing mitochondrial ROS and activities of electron transport chain complexes [34]. Thus, it is possible that under retrograde signaling is triggered by ROS, Ca^2+^, AMP/ATP and NAD^+^/NADH ratio in cells under oxidative stress. This further stimulates transfer of mitochondria from MSCs to recipient cells. In another study it was shown that calcium dependent mechanism of CD38/cyclic ADP ribose signaling mediated transfer of mitochondria from neurons to astrocytes for recycling and disposal of damaged mitochondria. This was seen to contribute to neuroprotective and neuro-recovery mechanism after stroke [46]. As CD38, catalyzes the synthesis of calcium messenger cyclic ADP-ribose in mitochondria membranes, its up regulation further increased mitochondria donation in a calcium dependent manner [47]. Another interesting study has shown that loss of cytochrome c in stressed cells, characterized by caspase 3 activation also triggered mitochondrial donation to rescue UV-damaged PC12 cells by MSCs in the early stages of apoptosis [48].

In addition, under stress, MSCs are programmed to secrete depolarized mitochondria for their own protection through transcellular mitophagy [49]. In an in vivo model of myocardial infarction, mitochondria released from damaged cells activated anti-apoptotic signals in MSCs. Foreign mitochondria from other cells were shown to be engulfed and degraded in MSCs, leading to induction of cytoprotective enzyme HO-1, and stimulation of mitochondrial biogenesis. This triggered enhanced mitochondrial donation from MSCs to these cells [40]. Phinney et al., have suggested that mitochondrial donation by MSCs is a protective mechanism adopted by MSCs rather than being an altruistic strategy [49]. Simultaneously, mitochondrial DNA that evades autophagy stimulates inflammatory responses mediated by Toll-like Receptor (TLR) 9 and other inflammatory responses, and eventually leads to cellular apoptosis [49–51]. Mitochondria proteins like TFAM and DAMPs including N-formyl peptides, mtDNA, cardiolipin or extracellular ATP are also known to participate in inflammatory responses and trigger transfer of mitochondria from donor to acceptor cells [52]. These studies have shown that various signaling molecules participate in mitochondria transfer from MSCs, but the exact mechanisms and choice of release of a particular signal molecule in a given situation followed by other downstream signals has not been well elucidated.

### Mechanisms of mitochondrial transfer

**Fig. 2:**
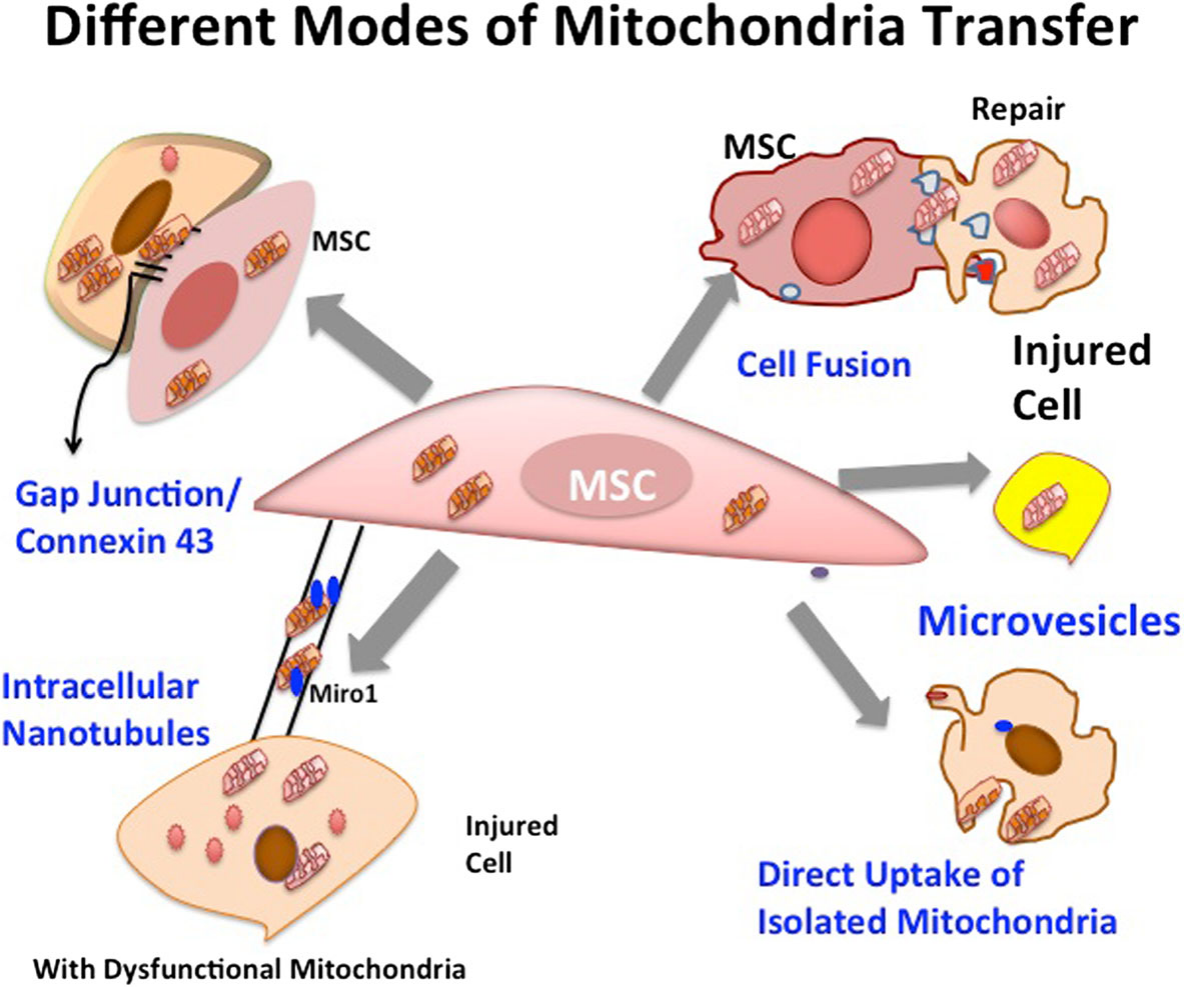
Different modes of mitochondrial transfer from MSCs to injured or damaged cells. These include transfer through intracellular nanotubes, gap junctions, cell fusion, microvesicles and direct uptake of isolated mitochondria

**Fig. 3:**
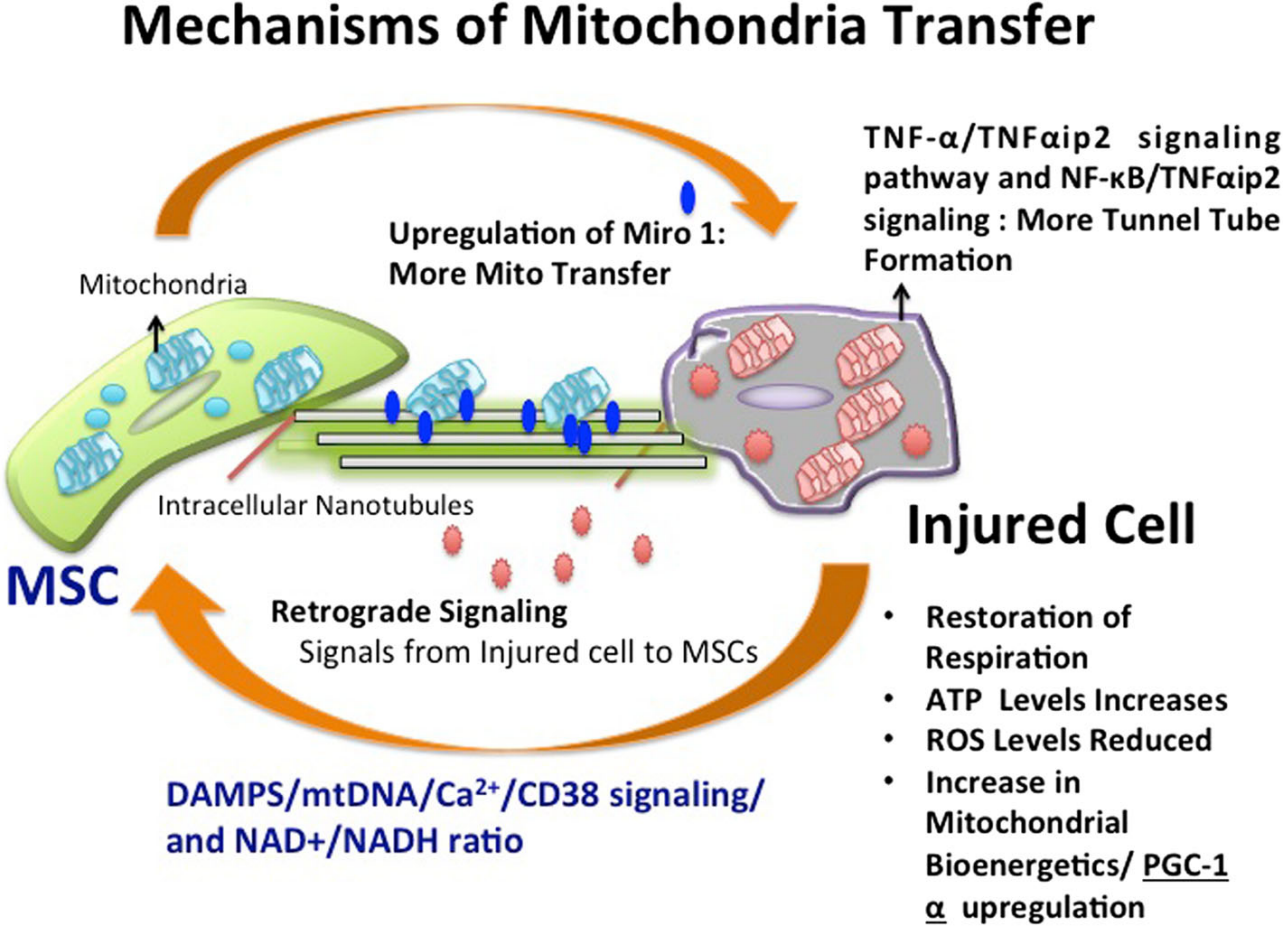
Mechanisms of Mitochondrial Transfer. An overview of mitochondrial transfer mechanisms has been shown with expression of Miro1 protein and tunnel tube formation signaling pathways along with signaling that trigger mitochondrial release from MSCs to injured cell under stress

Mitochondrial transfer has been found to be mediated by different modes that include tunnel tube formation, gap junctions, micro vesicles, cell fusion and transfer of isolated mitochondria [15, 34, 42, 53–56]. Figure 2 diagrammatically illustrates several modes of mitochondrial transfer from MSCs to recipient cells with damaged or dysfunctional mitochondria and Fig. 3 shows overview of mechanism involved in mitochondria trasnfer. A majority of studies illustrate mitochondrial transfer through formation of nanotubes between MSCs and damaged cells. Motor-adaptor protein complexes regulate mitochondrial transport and homeostasis, Miro 1 and Miro 2 are two dynamin related Rho-GTPases that interact with other accessory proteins to allow movement of mitochondria on cytoplasmic tunnel tube extensions that connect two cells. These calcium sensitive proteins bind mitochondria to KLF 5 kinesin motor protein with the help of other accessory proteins like TRAK 1 and TRAK 2, Myo 10 and Myo 19. Together, they form a motor-adaptor complex that contributes to mitochondria transport machinery and regulates movement of mitochondria on microtubules. The role of Miro1 was shown in nanotube transport in intercellular mitochondrial transport in a co-culture system of BM-MSCs and alveolar epithelial cells. MSCs overexpressing Miro1 were shown to perform enhanced rescue potential, leading to greater epithelial cell repair [34]. Down regulation of Miro1 expression and inhibition of tunnel tube formation prevented mitochondrial transfer from MSCs to epithelial cells. This study provided supporting evidence that established the significant role of Miro1 in mitochondrial transfer through tunnel tubes [34].

In an interesting study by Zhang et al., iPSCs show superior efficiency than BM-MSCs in mitochondrial transfer in rescue of Anthracycline induced cardiomyopathy due to higher expression of Miro 1 [41]. This was confirmed by various experiments showing that knockdown of Miro1 with short hairpin RNA (shRNA) that led to remarkable decrease in Miro1 expression and subsequent reduction in mitochondrial donation. Nonetheless, overexpression of Miro1 enhanced intracellular mitochondria transfer; thereby providing supporting evidence to confirm that mitochondria transfer is modulated by Miro1 expression. They also investigated tunnel tube formation between iPSCs and cardiomyocytes during mitochondrial transfer. It was shown that no mitochondria transfer occurred when cells were treated with cytochalasin B, that inhibits actin polymerisation and tunnel tube formation. These iPSCs were also found to be more responsive to tumor necrosis factor alpha (TNFα) that induced tunneling nanotube (TNT) formation in cardiomyocytes by elevating TNFαIP2 expression, which was further, regulated by TNF-α/NF-kappaB/TNFαip2 signaling pathway. A comparative analysis between iPSCs and BM-MSCs showed that iPSCs displayed higher expression of TNFαip2, and more number tunnel tube formation per cell followed by increased mitochondrial transfer. Thus, this study shows that higher expression of both Miro1 and TNFαip2 leads to more mitochondrial transfer and tunnel tube formation and imparts a cell with higher efficiency to transfer mitochondria as in case of iPSCs as compared to BM-MSCs [41].

Similar results have been observed in another study where MSCs transferred mitochondria to corneal epithelial cells through tunnel tube formation via NF-κB/TNFαip2 signaling. This was confirmed by western blot analysis of protein showing that enhanced levels of phosphorylated of NF-κB subunit p-IκB along with elevated levels of TNFα and TNFαip2 was markedly increased during tunnel tube formation. It was observed that NF-κB inhibitor SC-514 significantly attenuated tunnel tube formation thus; demonstrating the role of NF-κB mediated signaling mechanism in tunnel tube formation. Furthermore, the role of ROS signal in stimulating of tunnel tube formation was validated by using antioxidant scavenger, N-acetylcysteine (NAC) treatment that reduced ROS levels and concomitantly resulted in inhibition of tunnel tube formation and mitochondria transfer between MSCs and CECs [16].

Majority of the studies have shown that mitochondrial transfer is mainly mediated through tunnel tube formation and micro vesicles rather than gap junctions. This could be because most of the studies are carried out in in vitro co-culture system wherein tunnel tube formation is easily observed. However, connexin 43 containing gap junctions channels have been particularly well observed in in vivo acute lung injury mouse model [15]. Sinclair and colleagues have characterized and compared different modes of mitochondrial transfer from different types of MSCs to bronchial epithelial cells [57]. Complete abrogation of mitochondrial transfer was observed by blocking tunnel tube formation by cytochalasin B and inhibition of microvesicles by dynasore [57]. Cytoplasmic transfer was found unaffected by Gap26 inhibitor of connexin 43. However, a cocktail of inhibitors exhibited conspicuous attenuation of mitochondrial transfer and at greater extents suggesting synergy between these modes of mitochondrial transfer [57]. Uptake of mitochondria isolated from stem cells has also been observed in case of MDA-MB231 cell lines and cardiomyocytes [54, 58]. Although, the exact mechanism of mitochondrial uptake is yet unknown but few studies have suggested that internalization of mitochondria is mediated by actin-dependent cellular mechanisms [53, 58, 59]. Different modes of transfer have been observed under various pathophysiological conditions as illustrated further in this review. However, the basis for choice of mode of mitochondrial transfer under a particular condition is unclear. Though, it is quite possible that the proximity of damaged cells to MSCs and the environmental condition might act as contributing factors regulating the mode of transfer for mitochondrial transfer.

In a study by Spees et al., BM-MSCs were shown to transfer functional mitochondria to rhoφ cells devoid of functional mitochondria. Careful examination of these rescued cells excluded cell fusion as one of the main mechanisms of mitochondrial transfer. It was also shown that active formation of TNTs or vesicular transfer of mitochondria, but not passive uptake of mitochondrial fragments, resulted in functional complementation. Islam et al showed that formation of connexin-43 gap junction channels is essential for mitochondrial transfer from BM-MSCs to alveolar epithelial cells, thus increasing epithelial ATP production. BM-MSCs expressing dysfunctional connexin 43 could not adhere to alveolar epithelium, and also could not transfer mitochondria. Cytosolic calcium ions and cellular bioenergetics levels of ATP and glucose are also considered as major players in regulating movement of mitochondria [42]. Further comprehensive studies and elucidations of mitochondrial transfer mechanisms are required to better understand the signaling pathways and molecules involved in this process to employ mitochondrial transfer to many therapeutic applications.

### Mitochondrial transfer in regeneration of diseased tissues

Mitochondria transfer from MSCs has been studied in various disease models that have not only provided evidence for its significance in regenerative medicine but also helped to gain insight on mitochondria transfer mechanisms. Different preferred mechanism and modes have been observed on the basis of concerned recipient cells and stress condition. In lung cells, renal cells, corneal epithelial cells and brain cortical cells, mitochondria transfer has occurred mostly through tunnel tube formation. However, cell fusion and reprogramming of progenitor cells have been observed in cardiac cells. Micro vesicle formation has been observed predominantly in studies where immune-mechanisms are involved as in the case of anti-microbial activity. Nonetheless, remarkable restoration of cellular bioenergetics and reduction in oxidative stress was observed in all the studies that demonstrates that mitochondria transfer from MSCs plays critical role in cellular repair and regeneration. Also, another therapeutic strategy that targets inhibition of tunnel tube formation and mitochondrial transfer in case of tumors has also been discussed.

#### Lung diseases

Interestingly, mitochondrial transfer has been studied mostly in lung diseases. One of the remarkable works includes study carried out by Islam et al., wherein they found that BM-MSCs transfer mitochondria to pulmonary alveoli contributing to protection from acute lung injury [15]. This study reported mitochondrial transfer in intact lungs in in vivo mice model treated with lipopolysaccharide (LPS). It was noted that human or mice BM-MSCs instilled in mice airways were able to transfer mitochondria and repair mitochondrial bioenergetics in lungs. The mitochondrial transfer occurred over a period of 24 h in the lung epithelium and was supported by the expression of connexin 43 that mediated mitochondrial transfer through nanotubes and microvesicles in a calcium dependent manner. Mitochondrial transfer led to increased ATP concentrations in the recipient cells suggesting its role in bioenergetics. This study was followed by another remarkable discovery of role of Miro1, a mitochondrial Rho-GTPase that regulated mitochondrial transfer from MSCs to lung epithelial cells through connecting nanotubes [34]. In this study, Ahmad et al., reported that over expression of Miro1 enhanced mitochondrial transfer whereas its knockdown lead to the loss of rescue efficacy of MSCs. This study was performed in rotenone induced lung injury mouse model and further confirmed in allergen induced asthma models. Another study investigated the effect of mitochondrial transfer in rat models exposed to cigarette smoke for 56 days that induced lung damage and manifestation of chronic obstructive pulmonary disease [60]. It was reported that BM-MSCs mitigated the damage by transferring mitochondria to lung epithelium. Similar to results of previous studies, they also found significant mitochondrial transfer occurred after 24 h of MSC and BEAS-2B co-culture followed by subsequent elevation in ATP levels through tunnel tube formation. However, further research along these lines is warranted as the reasons for difference are vastly unknown and a comparative analysis of mitochondrial transfer abilities of stem cells from different sources remain unexplored. Li et al., have also suggested that mitochondrial dysfunction is observed in case of prolonged inflammation and stem cells can transfer mitochondria to mitigate inflammation indicative of their rescue potential by promoting anti-inflammatory effects.

#### Cardiac tissues

**Fig. 4:**
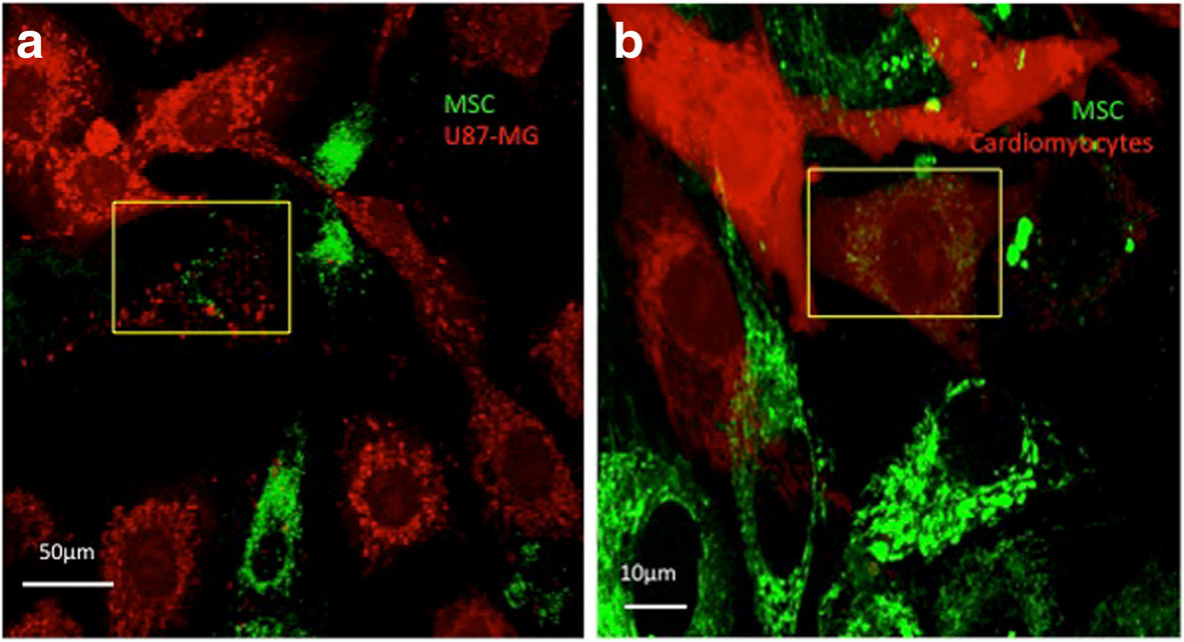
Mitochondria stained with MitoTracker Green FM (Thermo Fisher Scientific) in human BM-MSCs were transfered to Antimycin A treated (**a**) U87-MG and (**b**) rat cardiomyocyte. Confocal imaging was done on Leica TCS SP5 using software LAS AF

Mitochondrial transfer between endothelial progenitor cells were reported to regenerate and repair damaged myocardium [53, 61]. Vallabhaneni et al, reported the role of functional mitochondria transfer and intercellular communication between MSCs and vascular smooth muscle cells through intercellular tunnel tube bridges and not necessarily through differentiation, as claimed by earlier studies [62]. This study was also validated in another investigation that demonstrated cell-to-cell connection between endothelial cells and cardiomyocytes leading to mitochondrial transfer through nanotube formation, independent of cellular or nuclear fusion [35, 53, 63]. Cellular processes involved in mitochondrial dynamics play critical role in cardiovascular systems [64]. Dysfunctional mitochondria are predominant in cardiac disorders such as ischemia, myocardial infarction and other cardiomyopathies are found to be ameliorated by administration of healthy mitochondria [65–68]. Acquistapace et al., co-cultured human adipose stem cells with mouse cardiomyocytes and confirmed formation of intercellular F-actin connections showing mitochondrial transfer and partial cell fusion as mechanism of cell repair [33]. Cross-talk between stem cells and cardiomyocytes through tunnel tubes was further confirmed by other studies suggesting mitochondrial transfer as futuristic therapeutic strategy [17, 67, 69]. Figure 4 shows transfer of mitochondria stained with MitoTracker Green from human BM-MSCs to U87-MG cell line and human cardiomyocytes through tunnel tubes stained with phalloidin red. The cardiomyocytes were treated with antimycin A (100 μM) to introduce oxidative stress condition. Laser-patterned chips have also been devised that further shed light on mitochondrial transfer and cellular contact between MSCs and cardiomyocytes [70]. These studies have shown that mitochondrial transfer from stem cells can prove to be an effective therapeutic strategy to treat several cardiomyopathies.

#### Corneal epithelium and renal tubular cells

MSCs were found to transfer functional mitochondria to human corneal epithelial cells, which improved mitochondrial bioenergetics in recipient cells, studied using extracellular flux analyzer. Comparison of ATP production, resting respiration and maximal respiration rates showed that rates of these bioenergetics parameters were found to be lower in rotenone-treated corneal epithelial cells (CECs) as compared to normal CECs. All these parameters were improved in CECs co-cultured with MSCs. It was demonstrated that mitochondria were transferred from MSCs to damaged CECs, which also protected them from rotenone-induced cell death and proliferation-inhibition [16]. This study suggests that mitochondrial transfer can have huge implications in treating alkali-eye burns and other eye related damages. It can further prove to be an efficient strategy to provide protection to cornea against oxidative stress-induced mitochondrial damage. Interestingly, this study has also suggested a two-way flow of cytoplasmic components between cells that trigger the response of stem cells to the differentiated cells. Intercellular communication between renal epithelial cells has also been shown through exchange of cytoplasmic materials [71]. Mitochondrial transfer from MSCs to renal tubular cells has been observed in a co-culture of rat renal tubular cells and human MSCs through intercellular connections [72].

#### Brain cortical cells

Intercellular communication and mitochondrial transfer from MSCs to rat cortical neurons has been observed in co-culture in in vitro system [73]. A prominent neuroprotective behavior of MSCs was established through mitochondrial transfer and overexpression of Miro1, followed by subsequent increase in brain derived neurotrophic factor. Furthermore, intravenous injection of MSCs post ischemia was found to mitigate the pathological symptoms of stroke and restoration of neurological activity [73]. The role of mitochondrial transfer in brain cells is also evident from a recent interesting study that has shown transfer of mitochondria from astrocytes to neurons as a means of neuroglia cellular cross-talk in alleviation of stroke [47]. There is a pressing need to further investigate the role of mitochondrial transfer from stem cell to brain that can aid in devising better therapeutic strategies.

WJ-MSCs were co-cultured with mitochondrial DNA-depleted cells. Importantly, these cells regained the expression of mtDNA-encoded proteins and showed functional oxygen consumption and respiratory control, as well as the activity of electron transport chain complexes I, II, III and IV. In addition, metabolic shifting and ETC complex V-inhibitor-sensitive ATP production were also recovered. Furthermore, cellular behaviors like attachment-free proliferation, aerobic viability and OXPHOS-dependent cellular motility were also recovered after mitochondrial transfer by WJMSCs [74]. This study, along with the others cited above, provide strong evidence that MSCs can serve as potential therapeutics to ameliorate diseases with mitochondrial dysfunction, through donation of healthy mitochondria.

#### Antimicrobial activity of MSCs through mitochondrial transfer

MSCs are known to aid in regeneration through multiple ways that include anti-oxidant property, anti-inflammatory activities of MSCs and differentiation to regenerate damaged tissue. MSCs also demonstrate ability to combat infection by transferring mitochondria to macrophages. This leads to modulation of phagocytic ability of macrophages through tunnel tube by enhancing their bioenergetics evidenced by increased ATP activity. This facilitates more efficient clearing of bacterial cells, thereby influencing innate immune response of these cells serving as an important mechanism in pre-clinical models of acute respiratory distress syndrome (ARDS) and sepsis [75]. To establish that anti-microbial effect of MSCs is mediated by alveolar macrophage, effect of MSCs was determined in normal *E. coli* infected model with animal model treated with intranasal Clodronate Lipososmes (CL) that completely abrogated alveolar macrophages (AM). It was found that administration of MSC treatment in AM depleted mice was not able to restore levels of several cytokines involved in anti-inflammatory affects. But, in contrast MSCs could restore levels of cytokines in normal mice suggesting that the anti-microbial activity of MSCs is mediated through macrophages. Interestingly, macrophage phagocytosis of MSCs or fusion of MSC and macrophage was not observed in monocytes or neutrophils. This suggests that mitochondria transfer as the only means for enhanced engulfment by macrophages by both confocal and flow cytometry analysis. It was also observed that non-contact transfer of mitochondria through microvesicles or exosomes significantly enhanced mitochondria transfer and phagocytosis index of macrophages suggesting it as another critical mechanism for mitochondrial transfer. Another study by Phinney et al., has also shown that mitochondria transfer through microvesicles to macrophages and support paracrine and immune reaction. However, further investigation to better understand the role of mitochondria in anti-microbial and immune-modulation can provide better insights of multi-level reparative contributions of mitochondria in maintenance of cellular health post transfer to recipient cells.

#### Inhibition of mitochondrial transfer: A potential target for anti-tumor therapies

Mitochondria are the key regulators of cellular bioenergetics and metabolism that greatly impacts cancer progression [27]. Many researchers have pointed out mitochondria as a potential target for cancer therapies [7, 76]. In terms of MSCs, many studies have reported transfer of mitochondria through stem cells to tumor cells via tunnel tube formation contributing to their chemo resistance and proliferation [18, 43]. A very interesting study by Dong et al., have demonstrated that metastatic melanoma cells without mitochondrial DNA and with defective respiratory function were unable to form tumours. However, upon acquiring intact mitochondria along with their mtDNA from their host through horizontal transfer, the cellular respiration is restored. This was shown to be critical in tumor generation in mice [77]. It has been found that mitochondrial transfer from bone marrow stem cells renders survival advantage to acute myeloid leukemic cells thereby making them resistant to chemotherapy [18]. In a recent study, it was shown that inhibition of ICAM-1 an adhesion molecule prevented mitochondria transfer from MSCs to Jurkat Cells treated with chemotherapeutic drug. This prevented tunnel tube formation and stopped transfer of mitochondria from MSCs to Jurkat cells and eventually induced chemotherapy induced cell-death [74]. Thus, inhibition of mitochondrial transfer can serve as a potential therapy target for cancer, such as selective blockage of tunnel tube formation for preventing mitochondrial transfer [11, 78]. However, in view of the importance of aerobic glycolysis in cancer cells it remains possible that altering mitochondrial transfer to cancer cells may have context dependent effects and this merits further investigation [79].

### Challenges and future scope

Mitochondrial dysfunction is usually linked to a variety of diseases and as discussed above, mitochondrial transfer from healthy MSCs can potentially serve as an effective remedy for alleviation of such diseases. Despite significant advancements in this field, mitochondrial transfer has yet not become a therapeutic strategy for treatment purposes. The optimization of MSC donor type, dose, and development of cell delivery techniques present unexplored challenges for medical intervention to treat pathological conditions with mitochondrial dysfunction. In addition, very few research groups have investigated if mitochondrial transfer is self-sufficient for repair or requires synergistic release of other paracrine effects such as immunomodulatory factors and exosomes. It is critical to analyze mitochondria transfer holistically by comparing roles of secretome, exosome and mitochondrial transfer acting together and in isolation to better understand the regenerative mechanisms. Ahmad et al., demonstrated that paracrine mediators such as NO, TGF**-**β, IL-10 and PGE2 were unaffected by Miro1 overexpression, suggesting that mitochondrial transfer can work independent of paracrine effects [34]. In another study by Zhang et al., it was suggested that both mitochondrial transfer and paracrine factors provide protective advantage to cardiomyocytes against stress conditions. But, both these processes work independent of each other [41]. MSCs were seen to manage intracellular oxidative stress by targeting depolarized mitochondria to the plasma membrane via microvesicles. These are then taken up by macrophages, resulting in enhanced bioenergetics in recipient cells. An orchestra of proteins are then activated by release of miRNA from exosomes that further de-sensitize uptake of mitochondria suggesting a cross-talk between mitochondria and other paracrine factors [75]. But, combinatorial effect of these mechanisms in other models is largely unexplored and a systematic study is important to completely understand these mechanisms and their synergy.

## Conclusion

Mitochondrial transfer through MSCs has shown promising therapeutic results in varied disease conditions through various modes of action as discussed in this review. In addition, it is widely known that intercellular organelle communication aids in maintenance of cellular homeostasis, particularly in terms of energy metabolism and cellular bioenergetics regulation [56, 71, 80]. Several environmental cues such as oxidative stress, release of DAMPs and mtDNA from injured cell trigger mitochondria transfer from MSCs. This suggests that modulation of the extracellular milieu can regulate mitochondrial transfer that is evident from enhanced mitochondrial transfer from BM-MSCs upon treatment of anti-oxidants such as of N-acetyl-L-cysteine (NAC) and L-ascorbic acid 2-phosphate (AAP) [81]. Such innovative strategies can further be employed for efficient treatments using mitochondrial transfer. Furthermore, inhibition of mitochondrial transfer to tumor cells can serve as a novel strategy for cancer treatment [7, 18]. The use of isolated and artificial mitochondria for treatment of diseases appears to be a lucrative future therapy [55]. Further investigations are imperative to use artificial mitochondria for treatment of diseases. The optimum dose, packaging, delivery methods and ethical issues for using isolated mitochondria have yet not been studied. It is interesting to note that isolated mitochondria may emerge as an off-the shelf therapy in future, as many researchers are now exploring the role of isolated mitochondria in regenerative medicine.

## Abbreviations

AAP: ascorbic acid 2-phosphate
AD-MSCs: adipose derived stem cell
AM: alveolar macrophages
ARDS: Acute Respiratory Distress Syndrome
BM-MSCs: Bone marrow mesenchymal stem cell
CL: Clodronate Lipososmes
DAMPS: damage associated molecular patterns
DP-MSCs: Dental Pulp derived mesenchymal stem cell
iPSCs: Induced Pluripotent Stem Cells
MSCs: Mesenchymal stem cells
WJ-MSCs: Wharton’s Jelly, mesenchymal stem cell
